# MAGI Proteins Regulate the Trafficking and Signaling of Corticotropin-Releasing Factor Receptor 1 via a Compensatory Mechanism

**DOI:** 10.5334/1750-2187-11-5

**Published:** 2016-11-28

**Authors:** Maha M. Hammad, Henry A. Dunn, Stephen S. G. Ferguson

**Affiliations:** Department of Physiology and Pharmacology, University of Western Ontario, London, Ontario N6A 5B7, Canada; University of Ottawa Brain and Mind Institute and Department of Cellular and Molecular Medicine, University of Ottawa, 451 Smyth Dr. Ottawa, Ontario K1H 8M5, Canada

**Keywords:** corticotropin-releasing factor receptor 1, MAGI, compensatory mechanism, signaling, trafficking, PDZ protein interaction

## Abstract

Corticotropin-releasing factor (CRF) receptor1 (CRFR1) is associated with psychiatric illness and is a proposed target for the treatment of anxiety and depression. Similar to many G protein-coupled receptors (GPCRs), CRFR1 harbors a PDZ (PSD-95/Disc Large/Zona Occludens)-binding motif at the end of its carboxyl-terminal tail. The interactions of PDZ proteins with GPCRs are crucial for the regulation of receptor function. In the present study, we characterize the interaction of all members of the membrane-associated guanylate kinase with inverted orientation PDZ (MAGI) proteins with CRFR1. We show using co-immunoprecipitation that CRFR1 interacts with MAGI-1 and MAGI-3 in human embryonic kidney (HEK293) cells in a PDZ motif-dependent manner. We find that overexpression as well as knockdown of MAGI proteins result in a significant reduction in CRFR1 endocytosis. This effect is dependent on an intact PDZ binding motif for MAGI-2 and MAGI-3 but not MAGI-1. We show that the alteration in expression levels of MAGI-1, MAGI-2 or MAGI-3 can interfere with β-arrestin recruitment to CRFR1. This could explain the effects observed with receptor internalization. We also find that knockdown of endogenous MAGI-1, MAGI-2 or MAGI-3 in HEK293 cells can lead to an enhancement in ERK1/2 signaling but has no effect on cAMP formation. Interestingly, we observe a compensation effect between MAGI-1 and MAGI-3. Taken together, our data suggest that the MAGI proteins, MAGI-1, MAGI-2 and MAGI-3 can regulate β-arrestin-mediated internalization of CRFR1 as well as its signaling and that there is a compensatory mechanism involved in regulating the function of the MAGI subfamily.

## Introduction

Membrane-associated guanylate kinase (MAGUK) family proteins are synaptic scaffolding proteins within a structured protein network responsible for the spatial organization of the presynaptic and postsynaptic compartments. They play a crucial role in the formation and function of synapses in the central nervous system (CNS) by regulating multiple aspects of synapse physiology such as synaptogenesis, receptor trafficking, synaptic function, and plasticity [1,2,3]. MAGUKs are well-conserved throughout evolution and are widely expressed in the brain and periphery. They include multiple subfamilies including membrane palmitoylated proteins (MPPs), zona occludens (ZO), caspase recruitment domain-containing MAGUK protein (CARMA), discs large (DLGs) and MAGUK with inverted orientation PSD-95/Disc Large/Zona Occludens (PDZ) (MAGI) proteins [4]. Generally, these proteins contain multiple domains that control their function and facilitate their interactions with their targets. The two common domains among all members are PDZ domains and the guanylate kinase (GK) domain [4]. An important target for the MAGUKs scaffolding proteins is the G protein-coupled receptors (GPCRs) family [5]. Many GPCRs encode a short class I PDZ-binding motif (S/T-x-ϕ, where ϕ is any aliphatic amino acid residue) at the end of their carboxyl-terminal tail that is recognized by the PDZ domain of the MAGUK proteins. This protein-protein interaction between the receptor and MAGUKs results in the regulation of GPCR function. MAGUK proteins play a key role in mediating the subcellular localization, trafficking, cell surface expression and signal transduction of multiple GPCRs and different proteins have both overlapping and distinct roles in the regulation of GPCR activity [6,7,8,9]. One of the important subfamilies of MAGUKs is the membrane-associated guanylate kinase with inverted orientation (MAGI) protein subfamily [5], which consists of three members; MAGI-1, MAGI-2 and MAGI-3. All three proteins share a similar structure containing one guanylate kinase-like (GK) domain, two tryptophan tryptophan (WW) domains and six PDZ domains. MAGI proteins, particularly MAGI-2 and MAGI-3, have been shown to regulate the trafficking and signaling of multiple GPCRs.

MAGI-1, also known as BAP-1 (BAI-1-associated protein 1), has 7 isoforms that are widely expressed in different tissues. Isoform 1, isoform 2 and isoform 6 are highly expressed in colon, kidney, lung, liver, and pancreas. Isoform 5 is predominantly expressed in brain and heart. Isoform 3 and isoform 4 are highly expressed in pancreas and brain [10,11]. MAGI-1 interacts with BAI-1 (brain-specific angiogenesis inhibitor-1), a family B GPCR that functions as an adhesion molecule. Therefore, it is suggested that MAGI-1 might play an important role in the organization of membrane proteins and cytoskeleton by transmitting signals related to cell- cell or cell- matrix interactions through BAI-1 [12,13,14]. MAGI-1 also regulates AMPA receptor activity and modulate behavioral plasticity [15].

MAGI-2, also known as S-SCAM (synaptic scaffolding molecule), is specifically expressed in the brain and has 2 isoforms [16]. A previous study has illustrated an association between MAGI-2 and β_1_-AR that is enhanced upon agonist stimulation [17]. The study also reports an increase in agonist-induced internalization of β_1_-AR when MAGI-2 is co-expressed, but no effect on cAMP generation induced by isoproterenol stimulation. In contrast, MAGI-2 inhibits both vasoactive intestinal polypeptide type 1 (VPAC_1_) receptor-mediated cAMP production and internalization [18].

MAGI-3 is also widely expressed in different tissues. It has been shown to interact with β_2_-AR and reduce signaling via the extracellular signal-regulated kinase 1 and 2 (ERK1/2) pathway [19]. MAGI-3 also associates with lysophosphatidic acid-activated receptor subtype-2 (LPA_2_R) and opposite to the effect that is observed for the β_2_-AR, knockdown of MAGI-3 results in a decrease in LPA_2_R-mediated ERK 1/2 signaling [20]. The study also reports a significant reduction in Rho activation upon knockdown of MAGI-3.

The corticotropin-releasing factor receptor 1 (CRFR1) is activated by corticotropin-releasing factor (CRF), a 41 amino acid neuropeptide secreted from the paraventricular nucleus of the hypothalamus in response to stress. Release of CRF can lead to the activation of the hypothalamic-pituitary-adrenal axis (HPA axis) [21,22]. CRF can also bind to and activate CRFR2, however the affinity of CRF for CRFR1 is much higher than that for CRFR2. Both CRF receptors are widely expressed in the brain (neocortex and cerebellum) and pituitary [23,24]. A number of studies have elucidated a link between the pathophysiological changes in the CRF system and various neuropsychiatric disorders such as major depression, panic disorder, anorexia nervosa, and Alzheimer’s disease [25,26]. CRFRs can primarily couple to Gα_s_ for the activation of adenylyl cyclase leading to the formation of cyclic adenosine monophosphate (cAMP) [27] as well as the mitogen-activated protein kinase (MAPK) signaling pathway [28]. CRFR1 encodes a PDZ-binding motif at the carboxyl-terminal tail presented by the last three amino acids, threonine – alanine – valine (TAV). Our laboratory has reports of the interaction between CRFR1 and multiple PDZ domain-containing proteins including SAP97, PSD-95, CAL and PDZK1. These studies showed that both SAP97 and CAL function to antagonize the internalization of CRFR1 [29,30]. Interestingly, the two proteins had opposite effects on CRF-stimulated ERK1/2 signaling where knockdown of SAP97 suppressed ERK activation while knockdown of CAL resulted in a significant enhancement of this activation. CAL, a Golgi PDZ protein, seems to regulate CRFR1 function via mediating the post-translational modifications since it prevents the glycosylation of the receptor in the Golgi. On the other hand, SAP97 regulation of ERK signaling is a general regulatory mechanism that is independent from the receptor-PDZ protein interaction. This only further confirms the distinct functions of PDZ proteins depending on the particular GPCR with which they are associated. PDZK1 had no significant effect on the endocytosis of CRFR1 but enhanced ERK1/2 signaling [31].

A PDZ overlay assay previously performed utilizing the carboxyl-terminal tail of the CRFR1 suggests an interaction between the first PDZ domain of all three MAGI proteins with the receptor [29]. Therefore, we have further investigated these interactions and examined the effects of the MAGI family members on CRFR1 signaling and trafficking. We find that all three MAGI proteins as MAGUK proteins can interact with CRFR1 via the class I PDZ-binding motif. We find that MAGI proteins can regulate the endocytosis of CRFR1 by mediating β-arrestin recruitment upon stimulation with CRF. We also demonstrate that siRNA knockdown of MAGI proteins can result in an enhancement in ERK1/2 signaling. Furthermore, we show that knocking down one of the MAGI proteins results in the upregulation of the expression levels of the other members of the MAGI subfamily suggesting a compensatory mechanism for regulation. Taken together, our results indicate that MAGI proteins interactions with CRFR1 play an important role in regulating CRFR1 function.

## Materials and Methods

### Materials

Protein G beads were purchased from GE Healthcare (Oakville, ON, Canada). CRF was purchased from R&D Systems (Minneapolis, MN). HA peroxidase high affinity antibody was purchased from Roche (Mississauga, ON, Canada). Rabbit anti-GFP antibody was obtained from Invitrogen/Life Technologies (Burlington, ON, Canada). MAGI-1, MAGI-2 and MAGI-3 antibodies were purchased from Thermo Fisher (Burlington, ON, Canada). ECL Western blotting detection reagents were purchased from Biorad (Mississauga, ON, Canada). Mouse anti-HA antibody and all other biochemical reagents were purchased from Sigma-Aldrich (Oakville, ON, Canada).

### Plasmids

HA-CRFR1, CRFR1-YFP and HA-CRFR1-ΔTAV were described previously [32,33]. His-MAGI-1, HA-MAGI-2 and His-MAGI-3 were kindly provided by Dr. Randy Hall (Emory University School of Medicine). MAGI-1 siRNA, MAGI-2 siRNA and MAGI-3 siRNA were purchased from Thermo Fisher (Burlington, ON, Canada). For the negative control, we used Silencer Negative Control #1 AM4635 AGUACUGCUUACGAUACGGTT from Thermo Fisher (Burlington, ON, Canada). The exchange proteins directly activated by cAMP biosensor (EPAC) was a gift from Drs. Ali Salahpour (University of Toronto) and Marc Caron (Duke University) [34].

### Cell Culture and Transfection

Human embryonic kidney (HEK293) cells were cultured in Eagle’s minimal essential medium supplemented with 10% fetal bovine serum. Cells were plated on 10-cm dishes 24 hour prior to transfection. All experiments were performed on 75–80% confluent plates. Transfections were performed using calcium phosphate protocol except for siRNA transfections which were performed using Lipofectamine 2000 following manufacturer’s instructions. Transfections were performed with 1 μg of each construct. Empty pcDNA3.1 vector was used to equalize the total amount of plasmid cDNA used to transfect cells. 18 hours post-transfection, cells were washed with phosphate-buffered saline (PBS) and resuspended with trypsin, 0.25% EDTA. Experiments were performed approximately 48 hours after transfection except for knockdown experiments which were performed 72 hours post transfection since this protocol resulted in the maximum knockdown of the different MAGI proteins.

### Co-immunoprecipitation

24 hours after transfection, HEK293 cells were seeded onto 10-cm dishes. Cells were starved with HBSS for 1 hour at 37°C then stimulated with 100 nM CRF agonist for 30 min. Cells were then lysed in 500 μL lysis buffer (50 mM Tris, pH 8.0, 150 mM NaCl, and 0.1% Triton X-100) containing protease inhibitors (1 mM AEBSF, 10 μg/ml leupeptin, and 2.5 μg/ml aprotinin) for 20 min on a rocking platform at 4°C. Samples were collected into 1.5 ml Eppendorf tubes and centrifuged at 15,000 g for 15 min at 4°C to pellet insoluble material. A Bradford protein assay was performed and 300 μg of protein was incubated overnight at 4°C with protein G-Sepharose and mouse anti-HA antibody (1:50). Beads were washed three times with cold lysis buffer and incubated overnight at room temperature in 3xSDS loading buffer containing 2-mercaptoethanol. Samples were separated by SDS-PAGE, transferred to a nitrocellulose membrane, and western blots were then performed with the indicated antibodies (rabbit anti-GFP, 1:10000), (HA-POD, 1:1000), (rabbit anti-MAGI-3, 1:1000), (rabbit anti-V5, 1:1000).

### Bioluminescent Resonance Energy Transfer-based biosensor cAMP Assay

HEK293 cells were co-transfected with 1 μg HA-CRFR1 (WT or ΔTAV) and either 1μg pcDNA3.1, MAGI-1, MAGI-2, MAGI-3, 80 pmoles scrambled (SCR) siRNA or siRNA against MAGI-1, MAGI-2 or MAGI-3 as well as 2 μg of an EPAC (exchange proteins directly activated by cAMP) construct in 10-cm dishes. 24 hours post-transfection, cells were reseeded into 96-well plate (~10,000 cells/well) and left for another 24 hours. On the following day, cells were serum-starved for 1 hour in induction buffer (200 μM isobutyl-1-methylxanthine (IBMX) in HBSS). Coelenterazine was then added to the wells at a final concentration of 5 μM. Cells were then stimulated with increasing concentrations of CRF peptide for 10 min. The plate was then read by a Victor Plate Reader (Perkin-Elmer) and the BRET signal was determined by calculating the ratio of the light emitted at 505 to 555 nm to the light emitted at 465 to 505 nm.

### Receptor Endocytosis

Following transfection, HEK293 cells were re-seeded into 12-well plates. Cells were serum-starved in HBSS for 1 hour at 37°C then stimulated for 0 or 30 min with 500 nM CRF in HBSS at 37°C. Cells were washed with cold HBSS and incubated with mouse anti-HA antibody (1:1000) for 1 hour on ice. Cells were washed with cold HBSS then labeled with Alexa Fluor 647 donkey anti-mouse IgG (1:1000) for 1 hour on ice in the dark. Cells were washed with cold HBSS and treated with 5mM EDTA in PBS for 5 min on ice. Cells were collected and transferred to flow cytometry tubes containing 4% formaldehyde in PBS. Samples were run on a FACS Calibur cytometer using BD Cell Quest Pro software (BD Biosciences, Mississauga, ON) until 10,000 cells were counted. The geometric mean of fluorescence was determined using Flow Jo analysis software and was representative of the expression levels of the receptor on the plasma membrane (BD Biosciences, Mississauga, ON).

### Western Blot Analysis

75–90 μl of the samples from the different assays which is equivalent to about 50–70 μg of protein was diluted in β-mercaptoethanol-containing 3x loading buffer and then applied to 10% SDS-PAGE (30% acrylamide mix, 1.5M tris-HCl, 20% SDS, 10% ammonium persulfate and TEMED). Separated proteins were then transferred to nitrocellulose membranes and membranes were then blocked in 10% milk in TBS for 1 hour. Membranes were then blotted overnight by incubation with the appropriate antibody at 4°C. 24 hours later, membranes were washed at least three times with 1X TBS with 0.05% Tween 20, and then incubated with a horseradish peroxidase-conjugated secondary antibody (1:10000) for 1 hour. Membranes were finally washed again with 1X TBS with 0.05% Tween 20 three times before being developed using a BioRad chemiluminescence system.

### Bioluminescent Resonance Energy Transfer

Cells were co-transfected with the indicated cDNA using Lipofectamine 2000. β-arrestin was tagged with Renilla luciferase (Rluc) and used as the energy donor while CRFR1 was tagged with YFP and used as the energy acceptor. 72 hours after transfection, cells were starved with HBSS for 1 hour at 37°C. The reaction was then started by the addition of Coelenterazine at a final concentration of 5 μM followed by increasing concentrations of CRF. Signal was collected on a Synergy Neo2 plate reader (Thermo Fisher) using 460/40-nm (luciferase) and 540/25-nm (YFP) band pass filters. Whether or not BRET occurred was determined by calculating the ratio of the light passed by the 540/25 filter to that passed by the 460/40 filter. This ratio is referred to as the BRET ratio.

### Statistical Analysis

All measurements are represented as mean ± SEM. Comparisons were performed using one way analysis of variance test (ANOVA) followed by Bonferroni’s or Dunn’s Multiple comparisons test to determine significance. * indicate p values less than 0.05 and is considered to be significant.

## Results

### MAGI proteins interact with CRFR1 via the PDZ-binding motif independent of CRF activation

**Figure 1:**
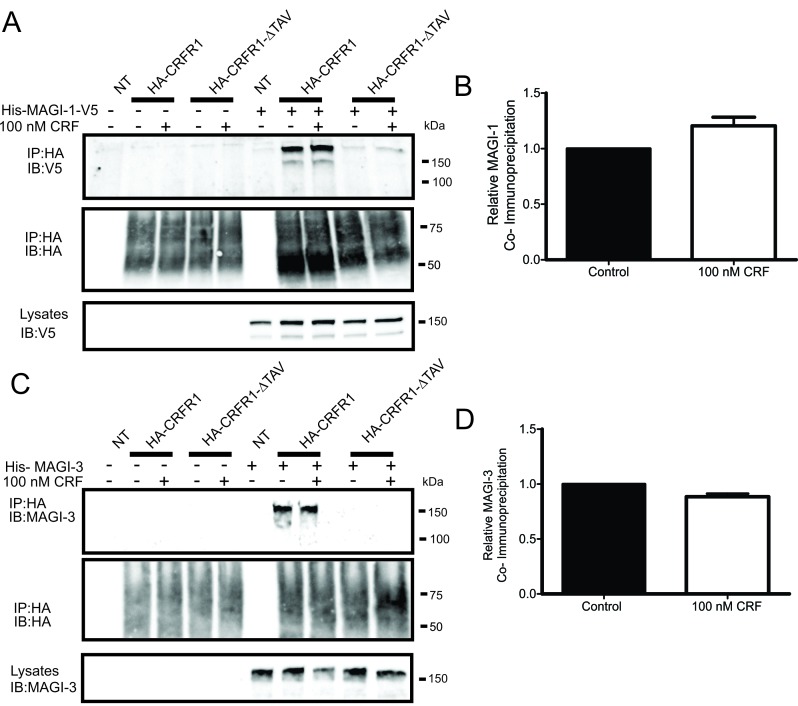
MAGI proteins co-immunoprecipitate with HA-CRFR1 in a PDZ-binding motif-dependent manner. HEK293 cells were co-transfected with HA-CRFR1 (WT or ΔTAV) and pcDNA or His-MAGI-1-V5. **(A)** Representative immunoblot of His-MAGI-1-V5 co-immunoprecipitated (IP) with HA-CRFR1 but not HA-CRFR1-ΔTAV which lacks the PDZ-binding motif. SDS-PAGE was used to analyze samples and immunoblots were performed with rabbit anti-V5. **(B)** Effect of CRF treatment was quantified using densitometry and had no significant difference on the amount of MAGI-1 co-immunoprecipitated with HA-CRFR1. HEK293 cells were co-transfected with HA-CRFR1 (WT or ΔTAV) and pcDNA or His-MAGI-3. **(C)** Representative immunoblot of His-MAGI-3 co-immunoprecipitated (IP) with HA-CRFR1 but not HA-CRFR1-ΔTAV which lacks the PDZ-binding motif. SDS-PAGE was used to analyze samples and immunoblots were performed with rabbit anti-MAGI-3. **(D)** Effect of CRF treatment was quantified using densitometry and had no significant difference on the amount of His-MAGI-3 co-immunoprecipitated with HA-CRFR1. Data are representative of three independent experiments.

The different members of the MAGI subfamily of proteins were previously shown to interact with different GPCRs. MAGI-2 was shown to interact with β_1_-AR, mGluR1a and VPAC_1_ while MAGI-3 was shown to interact with β_1_-AR, β_2_-AR, BAI-1 and LPA_2_R. We also previously identified the first PDZ domains of MAGI-1, MAGI-2 and MAGI-3 as positive hits among several PDZ domains that interact with a GST fusion of the CRFR1 carboxyl-terminal tail [29]. To further validate this interaction between MAGI proteins and CRFR1, we performed co-immunoprecipitation experiments with His-MAGI-1 or His-MAGI-3 and either wild-type HA-tagged CRFR1 (HA-CRFR1) or HA-tagged CRFR1 mutant that lacked the PDZ-binding motif (HA-CRFR1-ΔTAV) in transiently transfected HEK293 cells. We found that His-MAGI-1 and His-MAGI-3 co-immunoprecipitated with HA-CRFR1 but not CRFR1-ΔTAV (Fig. 1A and 1C) and that the interaction with HA-CRFR1 was not altered following agonist treatment with 100 nM CRF for 30 min (Fig. 1B and 1D). Similarly, MAGI-2 was previously shown to interact with CRFR1 via the PDZ-binding motif by another group [35].

### Effect of MAGI proteins expression on cAMP signaling

**Figure 2:**
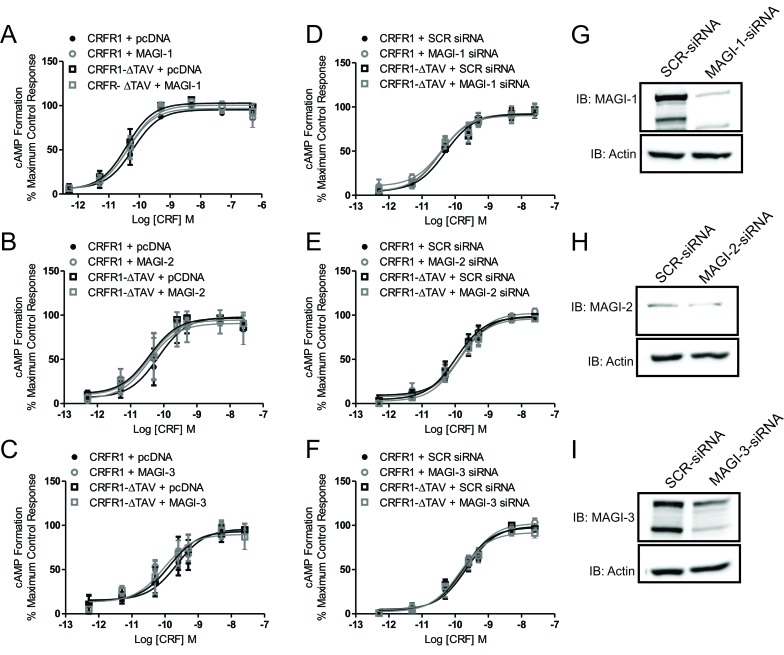
MAGI proteins do not regulate CRFR1-mediated cAMP formation. Dose response for CRF-stimulated cAMP formation assessed using a BRET-based cAMP biosensor assay. CRFR1- and CRFR1-ΔTAV- mediated cAMP formation in either plasmid vector or MAGI-1 **(A)**, MAGI-2 **(B)** or MAGI-3 **(C)** transfected HEK293 cells. CRFR1- and CRFR1- ΔTAV- mediated cAMP formation in either MAGI-1 siRNA **(D)**, MAGI-2 siRNA **(E)** or MAGI-3 siRNA **(F)** transfected HEK293 cells. Shown are representative immunoblots showing siRNA knockdown of **(G)** MAGI-1, **(H)** MAGI-2, and **(I)** MAGI-3 with 80 pmol siRNA for 72 hours. The data represent the mean ± SEM of at least three independent experiments.

PDZ domain-containing proteins were previously shown to have the potential to regulate cAMP signaling of some GPCRs [5,6] and MAGI-2 in particular was shown to reduce cAMP formation upon activation of VPAC_1_ [18]. Therefore, we tested whether MAGI proteins contributed to the regulation of CRFR1-mediated cAMP formation. In HEK293 cells transfected with either HA-CRFR1 or HA-CRFR1-ΔTAV, with or without MAGI-1, MAGI-2 or MAGI-3, there were no significant changes in the maximum efficacy for cAMP signaling in response to treatment with increasing concentrations of CRF for 10 min (Fig. 2A–C). In addition, we examined whether the attenuation of MAGI proteins expression could alter CRFR1-mediated cAMP signaling. We found that transfection of HEK293 cells with siRNA directed to knockdown MAGI-1, MAGI-2 or MAGI-3 had no effect on either HA-CRFR1- or HA-CRFR1-ΔTAV- stimulated cAMP signaling in response to treatment with increasing concentrations of CRF for 10 min (Fig. 2D–F). These results suggested that MAGI proteins did not contribute to the regulation of G protein-dependent signaling of CRFR1 presented by the activation of the cAMP pathway. We validated the knockdown of MAGI proteins by co-transfecting HEK293 cells with MAGI proteins cDNA (MAGI-1, MAGI-2 or MAGI-3) and 80 pmoles of either SCR siRNA or MAGI-siRNA. We observed 90% knockdown with MAGI-1 siRNA (Fig. 2G), 50% knockdown with MAGI-2 siRNA (Fig. 2H) and 65% knockdown with MAGI-3 siRNA (Fig. 2I).

### Effect of MAGI proteins on Cell Surface expression

**Figure 3:**
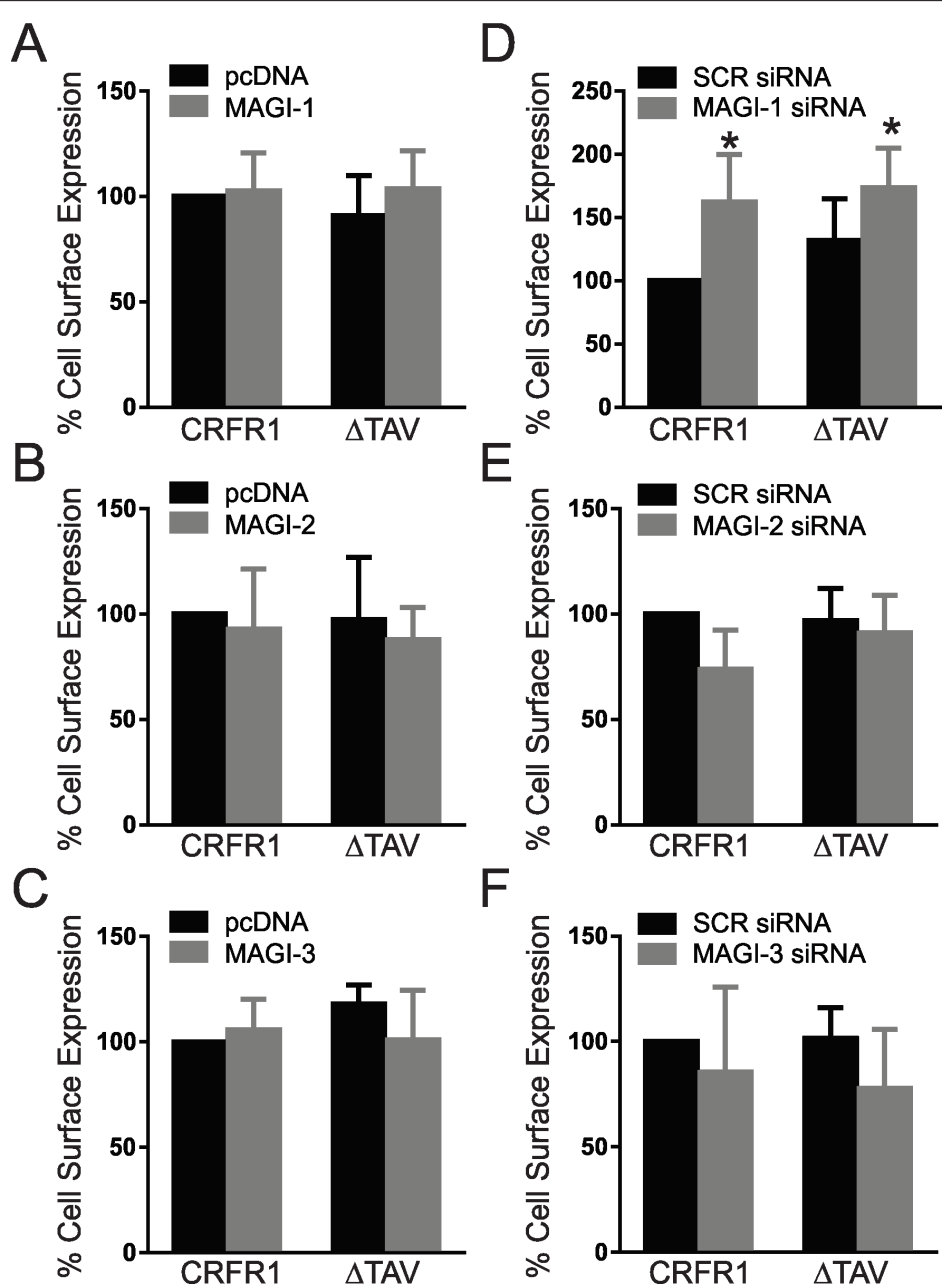
Effect of MAGI proteins on cell surface expression. Cell surface expression in HEK293 cells expressing either HA-CRFR1 or HA-CRFR1-ΔTAV along with MAGI-1 **(A)**, MAGI-2 **(B)**, MAGI-3 **(C)**, MAGI-1 siRNA **(D)**, MAGI-2 siRNA **(E)** or MAGI-3 siRNA **(F)** expressed as measured by flow cytometry. Data is normalized to HA-CRFR1 expression in the control pcDNA or SCR siRNA. The data represent the mean ± SEM of four independent experiments. P < 0.05 versus HA-CRFR1 cell surface expression.

A previous study from our laboratory illustrated an effect of CAL, a Golgi PDZ protein, on cell surface expression of CRFR1 [30]. Therefore, we used flow cytometry to measure the plasma membrane expression of CRFR1 upon overexpression as well as knockdown of MAGI proteins. While overexpression of MAGI proteins had no effect on receptor expression levels (Fig. 3A–C), knockdown of certain MAGI proteins altered CRFR1 membrane expression. Our data show that knockdown of MAGI-1 resulted in an enhancement in CRFR1 expression at the plasma membrane that was not dependent on the PDZ-binding motif (Fig. 3D). On the other hand, knockdown of MAGI-2 or MAGI-3 had no effect on CRFR1 expression levels. (Fig. 3E–F). This illustrates the importance of MAGI proteins in regulating receptor expression and reflects distinguished effects among the members of the subfamily.

### Effect of MAGI proteins on CRF-mediated ERK1/2 signaling

**Figure 4:**
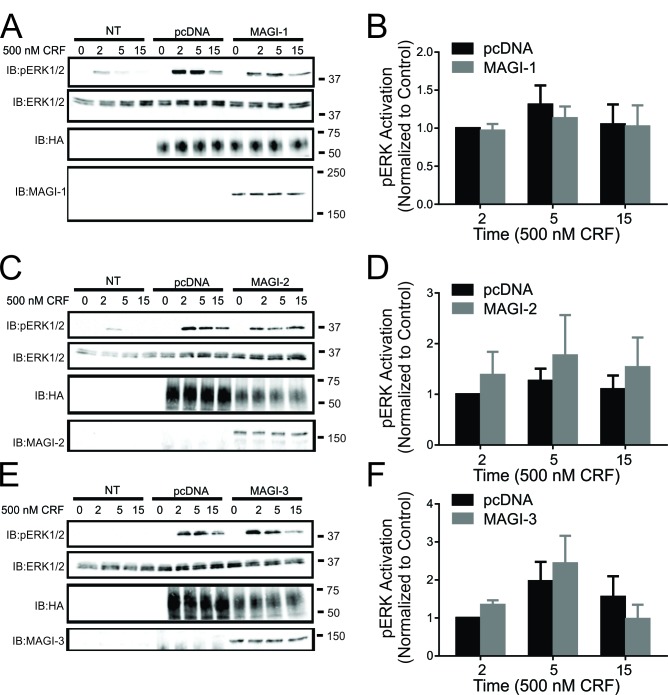
Effect of MAGI proteins overexpression on CRFR1-mediated ERK1/2 signaling. **(A)** Representative immunoblot showing ERK1/2 phosphorylation in response to 500 nM CRF treatment for 0, 2, 5, and 15 min in non-transfected (NT) HEK293 cells, and HEK293 cells transfected with HA-CRFR1 along with either pcDNA or His-MAGI-1. Also shown are corresponding immunoblots for total ERK1/2, MAGI-1 and HA-CRFR1 protein expression. **(B)** Densitometric analysis of ERK1/2 phosphorylation in response to 500 nM CRF treatment for 0, 2, 5, and 15 min. The data represent the mean ± SEM of four independent experiments. **(C)** Representative immunoblot showing ERK1/2 phosphorylation in response to 500 nM CRF treatment for 0, 2, 5, and 15 min in non-transfected (NT) HEK293 cells, and HEK293 cells transfected with HA-CRFR1 along with either pcDNA or MAGI-2. Also shown are corresponding immunoblots for total ERK1/2, MAGI-2 and HA-CRFR1 protein expression. **(D)** Densitometric analysis of ERK1/2 phosphorylation in response to 500 nM CRF treatment for 0, 2, 5, and 15 min. The data represent the mean ± SEM of six independent experiments. **(E)** Representative immunoblot showing ERK1/2 phosphorylation in response to 500 nM CRF treatment for 0, 2, 5, and 15 min in non-transfected (NT) HEK293 cells, and HEK293 cells transfected with HA-CRFR1 along with either pcDNA or MAGI-3. Also shown are corresponding immunoblots for total ERK1/2, MAGI-3 and HA-CRFR1 protein expression. **(F)** Densitometric analysis of ERK1/2 phosphorylation in response to 500 nM CRF treatment for 0, 2, 5, and 15 min. The data represent the mean ± SEM of six independent experiments.

**Figure 5:**
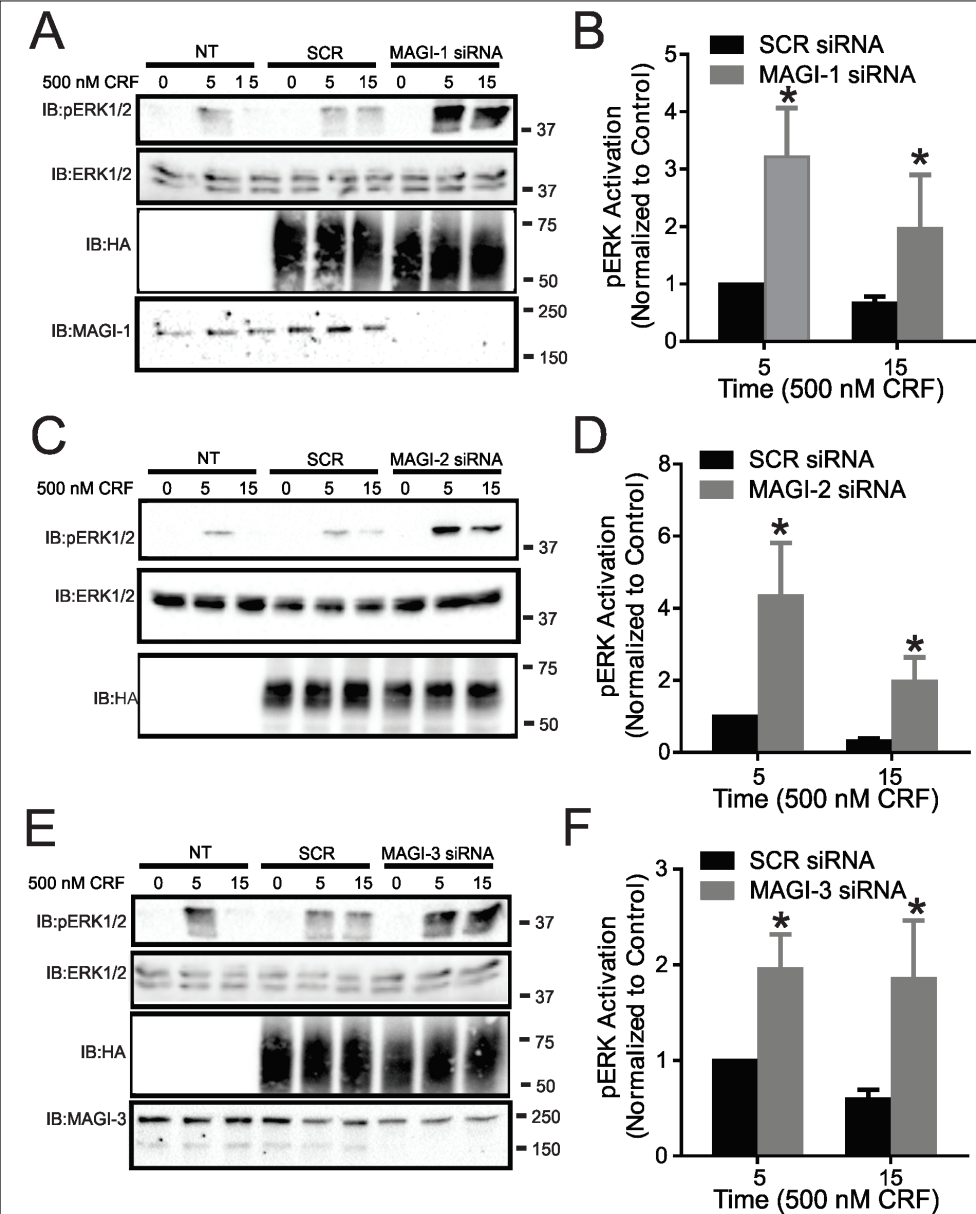
Effect of MAGI proteins knockdown on CRF-mediated ERK1/2 signaling. **(A)** Representative immunoblot showing ERK1/2 phosphorylation in response to 500 nM CRF treatment for 0, 5, and 15 min in non-transfected (NT) HEK293 cells, and HEK293 cells transfected with HA-CRFR1 along with either SCR siRNA or MAGI-1 siRNA. Also shown are corresponding immunoblots for total ERK1/2, MAGI-1 and HA-CRFR1 protein expression. **(B)** Densitometric analysis of ERK1/2 phosphorylation in response to 500 nM CRF treatment for 0, 5, and 15 min. The data represent the mean ± SEM of four independent experiments. **(C)** Representative immunoblot showing ERK1/2 phosphorylation in response to 500 nM CRF treatment for 0, 5, and 15 min in non-transfected (NT) HEK293 cells, and HEK293 cells transfected with HA-CRFR1 along with either SCR siRNA or MAGI-2 siRNA. Also shown are corresponding immunoblots for total ERK1/2 and HA-CRFR1 protein expression. **(D)** Densitometric analysis of ERK1/2 phosphorylation in response to 500 nM CRF treatment for 0, 5, and 15 min. **(E)** Representative immunoblot showing ERK1/2 phosphorylation in response to 500 nM CRF treatment for 0, 5, and 15 min in non-transfected (NT) HEK293 cells, and HEK293 cells transfected with HA-CRFR1 along with either SCR siRNA or MAGI-3 siRNA. Also shown are corresponding immunoblots for total ERK1/2, MAGI-3 and HA-CRFR1 protein expression. **(F)** Densitometric analysis of ERK1/2 phosphorylation in response to 500 nM CRF treatment for 0, 5, and 15 min. The data represent the mean ± SEM of four independent experiments. ^*^P < 0.05 versus SCR siRNA control.

MAGI-3 was previously shown to regulate ERK1/2 signaling of other GPCRs in a distinctive manner as it was shown to enhance ERK1/2 activation in the case of LPA_2_R and BAI-1 and inhibit β-AR-stimulated ERK1/2 phosphorylation [19,20,36]. Since CRFR1 was shown to have the ability to activate the MAPK cascade, we assessed the effects of MAGI proteins overexpression on CRFR1-mediated ERK1/2 phosphorylation. We found that the overexpression of MAGI-1, MAGI-2 and MAGI-3 did not have an effect on ERK1/2 phosphorylation (Fig. 4). Interestingly, when we knockdown endogenous expression of MAGI-1, MAGI-2 or MAGI-3, we observed a significant increase in ERK1/2 phosphorylation in response to stimulation of CRFR1 with 500 nM CRF for 5 or 15 minutes (Fig. 5).

### Effect of MAGI proteins expression on agonist-stimulated CRFR1 endocytosis

**Figure 6:**
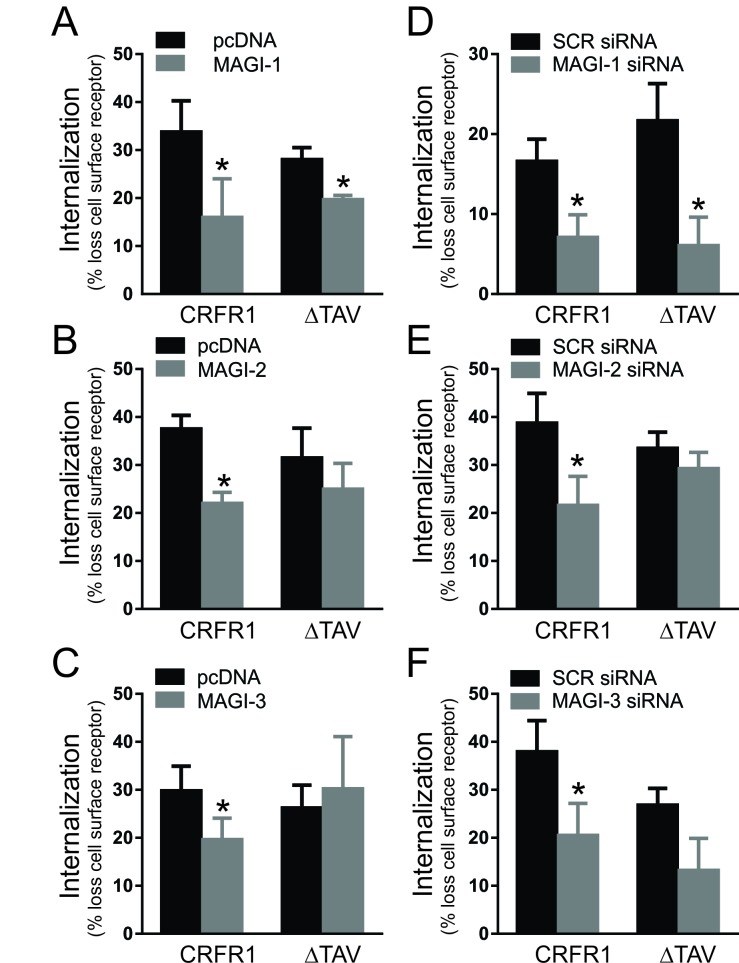
Effect of MAGI proteins expression on agonist-stimulated CRFR1 endocytosis. Agonist-stimulated internalization of either HA-CRFR1 or HA-CRFR1-ΔTAV following 500 nM CRF treatment for 30 min in the presence of either MAGI-1 **(A)**, MAGI-2 **(B)**, MAGI-3 **(C)**, MAGI-1 siRNA **(D)**, MAGI-2 siRNA **(E)**, or MAGI-3 siRNA **(F)**. The data represent the mean ± SEM of at least three independent experiments. * P < 0.05 versus empty vector or SCR siRNA control.

Our laboratory previously reported that other members of the MAGUK family such as SAP97 and PSD-95, as well as the Golgi PDZ protein CAL could regulate CRF-induced CRFR1 internalization [29,30,37]. To determine whether MAGI proteins also contributed to the regulation of CRFR1 endocytosis, we utilized flow cytometry to assess the loss of cell surface HA-CRFR1 by immunofluorescence. We found that the overexpression of MAGI-1, MAGI-2 and MAGI-3 each attenuated agonist-stimulated HA-CRFR1 internalization following treatment with 500 nM CRF for 30 min (Fig. 6A–C). However, although the internalization of the mutant HA-CRFR1-ΔTAV was not altered by either MAGI-2 or MAGI-3, the overexpression of MAGI-1 significantly reduced HA-CRFR1-ΔTAV (Fig. 6A–C). In addition, we assessed the effect of knockdown of endogenous MAGI proteins on agonist-stimulated CRFR1 internalization. We observe that all MAGI siRNAs attenuated the internalization of CRFR1 (Fig. 6D–F) and that MAGI-1 siRNA had the same effect on CRFR1-ΔTAV internalization (Fig. 6D). Thus, MAGI proteins are important for the regulation of CRFR1 endocytosis.

### MAGI proteins can regulate CRFR1 endocytosis by mediating β-arrestin recruitment to the receptor

**Figure 7:**
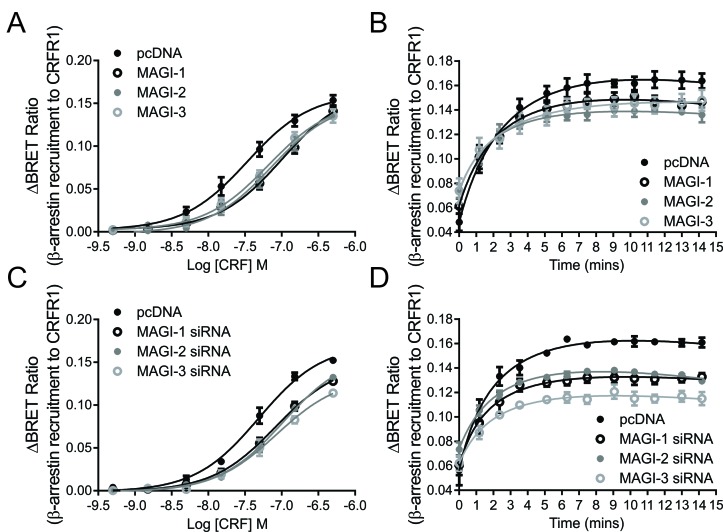
Effect of MAGI proteins expression on β-arrestin recruitment to CRFR1: BRET assays measured between β-arrestin-rLuc and CRFR1-YFP in the presence of MAGI-1, MAGI-2 or MAGI-3 **(A, B)** or MAGI-1 siRNA, MAGI-2 siRNA or MAGI-3 siRNA **(C, D)**. **A** and **C** show dose responses of the interaction while **B** and **D** show the interaction as a function of time upon stimulation with 500 nM CRF. The data represent the mean ± SEM of at least four independent experiments.

The primary pathway contributing to CRFR1 internalization was previously shown to be β-arrestin-dependent [32]. Because of the effect of MAGI proteins on the regulation of CRFR1 endocytosis observed in Fig. 6, we decided to study the effect of MAGI proteins on β-arrestin recruitment to CRFR1. To do this, we employed a BRET assay to investigate this interaction between rLuc-tagged β-arrestin2 and YFP-tagged CRFR1. The overexpression of all MAGI proteins resulted in a right-ward shift in the CRF dose response curve for β-arrestin2 recruitment (Fig. 7A). There was also a significant reduction in the maximal response for β-arrestin recruitment at 500 nM CRF with time (Fig. 7B). We also examined the effect of knockdown of MAGI proteins on β-arrestin2 recruitment and similar results were observed, with a right-ward shift in the does response curve and significant reduction in the maximal response, with MAGI-3 exhibiting the most prominent effect (Fig. 7C and 7D). Taken together, these data suggested that MAGI proteins are attenuating CRFR1 endocytosis by interfering with β-arrestin recruitment to CRFR1.

### Members of the MAGUK family of proteins can regulate the trafficking of GPCRs via a compensatory mechanism

**Figure 8:**
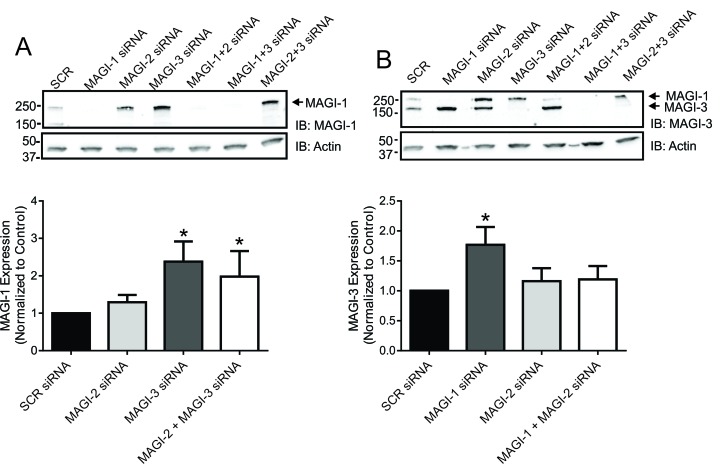
MAGUK proteins can regulate GPCRs function by a compensatory mechanism. **(A)** Representative immunoblot showing the levels of MAGI-1 in HEK293 cells transfected with siRNA combinations as labeled. Densitometric analysis shows MAGI-1 levels after normalization to actin as a loading control. **(B)** Representative immunoblot showing the levels of MAGI-3 in HEK293 cells transfected with siRNA combinations as labeled. Densitometric analysis shows MAGI-3 levels after normalization to actin as a loading control. The data represent the mean ± SEM of at least eight independent experiments. *P < 0.05 versus SCR siRNA control.

The results we obtained from our internalization assays and β-arrestin recruitment assays were surprising in terms of obtaining similar effects upon overexpression and knockdown of MAGI proteins. Therefore, we hypothesized that the expression levels of different members of the MAGI subfamily of proteins might be changing to compensate for the loss of another family member. In order to test this, we assessed the expression levels of each MAGI protein upon knockdown of the other MAGI proteins. We observed a significant up-regulation of MAGI-1 expression levels upon knockdown of MAGI-3 (Fig. 8A). Similarly, there was a significant up-regulation of MAGI-3 expression levels when MAGI-1 is knocked down (Fig. 8B). This suggested a compensatory effect between MAGI-1 and MAGI-3 that could explain the similar results observed for CRFR1 internalization and β-arrestin2 recruitment following either MAGI protein overexpression or knockdown.

## Discussion

We have previously identified all three members of the MAGI subfamily as CRFR1-interacting PDZ proteins in the PDZ overlay assay [29]. Despite similarities in their molecular topology, the members of the MAGI subfamily, specifically MAGI-2 and MAGI-3, are known to have distinct function with respect to their functional regulation of different GPCRs, but their role in regulating CRFR1 activity has not been investigated [17,18,19,20,38]. We find that all three MAGI proteins are co-immunoprecipitated with CRFR1 in a PDZ motif-dependent manner and that either overexpression or knockdown of each of the MAGI proteins is able to negatively regulate CRFR1 endocytosis with MAGI-1 overexpression or knockdown negatively regulating CRFR1 endocytosis in a PDZ motif-independent manner. This antagonism of CRFR1 endocytosis that is mediated by either MAGI protein overexpression or knockdown appears to involve the antagonism of β-arrestin recruitment to the receptor. In the case of siRNA-mediated knockdown of individual MAGI proteins, there appears to be a compensatory up-regulation of the expression of other MAGI protein subtypes. In addition we find that while the overexpression of each of the MAGI proteins does not influence CRFR1-stimulated ERK1/2 phosphorylation, siRNA knockdown of each of the MAGIs results in a significant increase in CRFR1-mediated ERK1/2 phosphorylation. Taken together, our results indicate that MAGI protein interactions with CRFR1 play an important role in regulating CRFR1 function.

MAGUK proteins comprise a subfamily of scaffolding proteins that are defined by having PDZ, SH3 and GK domains with an emerging role in synapse formation and function. DLG (discs large) subfamily (mainly SAP97 and PSD-95) have been the focus of many studies and are shown to play key roles in synaptogenesis, the creation of neural circuits, synaptic transmission, memory and learning, as well as glutamate receptor clustering and trafficking [13]. On the other hand, few studies have looked at the MAGI subfamily of MAGUK proteins, although they are abundantly expressed in the brain. Current understanding of MAGI protein function in the brain suggests an association with memory and learning, but the mechanism is still unknown [1,4,13]. In terms of the MAGUKs role in regulating GPCRs function, studies from our laboratory illustrate distinct roles for SAP97 and PSD-95 in regulating CRFR1 trafficking and signaling [29,37]. This is an intriguing observation because of the overlapping sequence and structural similarities between SAP97 and PSD-95. This lead to the hypothesis that MAGI proteins would also elicit diverse effects on receptor physiology. Although previous studies have looked at the effects of MAGI proteins on regulating GPCRs function, none of these previous reports have compared the relative functions of all three MAGI proteins in regulating the activity of an individual GPCR, despite evidence that many GPCRs interact with all three MAGIs [5,17,18,19,20,36,38]. For example, MAGI-2 interacts with the β_1_-AR and enhances its internalization, whereas it also interacts with VPAC_1_ to regulate its intracellular localization in epithelial cells and inhibit agonist-induced internalization [17,18,36]. MAGI-3 on the other hand is found to antagonize ERK1/2 activation by both the β_2_-AR and the LPA_2_R [19,20]. We find here that the overexpression of any of the MAGI proteins reduces CRF-stimulated CRFR1 internalization, which is similar to what is observed for VPAC_1_, but opposite to what is observed for the β_1_-AR. Interestingly, this effect is dependent on an intact CRFR1 PDZ-binding motif for MAGI-2 and MAGI-3, but not MAGI-1. This suggests that MAGI-1 may associate with CRFR1 via other interactions than those mediated by the PDZ domain. An alternative possibility would be that MAGI-1 is antagonizing the formation of the protein complexes required for GPCR endocytosis. Further studies on receptors that do not harbor a PDZ-binding motif such as CRFR2 would be required to further investigate this role of MAGI-1. In addition, PDZ proteins have been proposed to interact with β-arrestins to facilitate or prevent receptor internalization [39,40,41]. Thus, this association may prevent β-arrestin recruitment following PDZ protein overexpression. It will also be important to study the effect of MAGI-1 on the ESCRT complex and its components.

As is the case for many GPCRs, the primary internalization pathway utilized by the CRFR1 involves β-arrestin-dependent endocytosis via clathrin-coated pits [32,42,43,44]. The observation that each of the MAGI proteins antagonizes CRFR1 endocytosis suggested the possibility that MAGI proteins may play a role in regulating β-arrestin recruitment to the receptor. Consistent with this hypothesis, the overexpression of all of the MAGIs resulted in the antagonism of β-arrestin2 recruitment to the receptor as measured by BRET assay as assessed by a right-ward shift in the CRF dose response curve and a blunting of the maximal translocation response. Surprisingly, when the same assay is repeated with siRNA against MAGI-1, MAGI-2 or MAGI-3 to knockdown the endogenous proteins, we still observe a right-ward shift in the dose response curve and an even more prominent reduction in the maximal response. This result is opposite to what we had anticipated. Therefore, we investigated whether changes in the expression levels of other MAGI protein subtypes is compensating for the loss of expression of the MAGI protein knocked down by siRNA. This concept of compensation is not entirely novel to the field of PDZ-domain containing proteins, as it is suggested that PSD-95 and PSD-93 can replace one another to regulate GPCR function in a similar manner [45]. Interestingly, we observe a compensatory up-regulation of MAGI-1 expression upon MAGI-3 knockdown and an up-regulation of MAGI-3 expression following MAGI-1 protein knockdown. Thus, compensatory MAGI protein expression provides a plausible mechanism by which knocking down individual MAGI proteins attenuates CRFR1 endocytosis and impairs β-arrestin2 recruitment. However, this mechanism may not be entirely sufficient to explain our results, as although MAGI-2 knockdown resulted in increased MAGI-1 and MAGI-3 protein expression in the representative blots shown in Fig. 8, this result is not reliably reproduced when compared to MAGI-1 and MAGI-3 knockdown. Additional work would be necessary to further assess this interplay between the MAGI subfamily members.

We have reported previously the interaction of other PDZ-domain containing proteins with CRFR1 and the consequences of that on receptor endocytosis. We have shown that the overexpression of SAP97 antagonizes agonist-stimulated CRFR1 internalization, whereas single hairpin (shRNA) knockdown of endogenous SAP97 in HEK293 cells results in increased agonist-stimulated CRFR1 endocytosis and that PSD-95 elicits the same effects on CRFR1 endocytosis [29,37]. Similarly, we also find that CAL negatively regulates CRFR1 endocytosis when overexpressed [30]. Despite seemingly having similar effects, the mechanism of GPCR regulation by these PDZ proteins is vastly different. SAP97 and PSD-95 are suggested to regulate the internalization by affecting β-arrestin recruitment to CRFR1, while CAL acts as a major sorting protein at different subcellular levels and by modifying the post-translational modifications that the receptor undergoes at the Golgi. Thus, the MAGI proteins employ a similar “MAGUK-like” mechanism to regulate CRFR1 endocytosis via the attenuation of β-arrestin recruitment. However, their collective function is further complicated by a compensatory mechanism involving changes in protein expression of other subtypes. Future studies will examine this potential phenomenon and how it may affect other GPCRs function.

In addition to the role that MAGI proteins play in regulating the trafficking of CRFR1, we find that they have an important effect on the regulation of CRFR1 signaling, specifically via the MAPK pathway, but not G protein-mediated cAMP formation. While the overexpression of each of the MAGI proteins has no effect on CRFR1-mediated ERK1/2 phosphorylation, siRNA knockdown of any of the MAGIs results in a significant increase in the extent of ERK1/2 phosphorylation induced by CRFR1 activation. Interestingly, a study on LPA_2_R showed opposite effects on ERK1/2 activation where MAGI-3 knockdown reduced the signal. However, similar to our study, the LPA_2_R study which was performed on SW480 cells reported no difference in ERK activity upon overexpression of MAGI-3 suggesting that different tissues and cell lines express endogenous MAGI proteins in high levels. Previous experiments examining the regulation of CRFR1-mediated ERK1/2 activation by two other MAGUK family members, SAP97 and PSD-95 yielded different results [29,37]. Whereas, SAP97 expression significantly enhanced CRFR1-stimulated ERK1/2 phosphorylation, PSD-95 overexpression had no effect on ERK1/2 phosphorylation induced by CRFR1 activation. In contrast, the observed increase in CRFR1-mediated ERK1/2 phosphorylation following siRNA knockdown of each of the MAGI proteins is similar to what we have previously observed following siRNA knockdown of PDZK1 [31]. The mechanism by which siRNA knockdown of an individual MAGI protein results in compensatory changes in the expression levels of other MAGI proteins to attenuate CRFR1 endocytosis without compensating for CRFR1-mediated ERK1/2 activation is unclear. However, these results might suggest that the observed increase in ERK1/2 phosphorylation is mediated by a β-arrestin-independent pathway as CRFR1/β-arrestin interactions are predominantly regulated by GRK5 phosphorylation, which is thought to preferentially allow activation of ERK1/2 via the β-arrestin-mediated pathway [32,46].

Now that we have a better understanding for the role of MAGI proteins in regulating CRFR1 function in HEK293 cells, the next step would be to assess these interactions *in vivo*. RT-PCR in mouse tissue showed that the MAGI proteins are expressed in different regions of the brain including the cortex, thalamus and cerebellum [47]. We would predict that MAGI proteins are expressed in CRF-containing neurons and would regulate CRFR1 physiology. Some knockout mouse models of MAGI proteins are available (MAGI-1 and MAGI-2) and have exhibited some renal deficits [48]. This reflects the importance of MAGI proteins in periphery. In addition, MAGI-2 knockout mice show abnormal elongation of dendritic spines indicating a possible role for MAGI-2 during morphogenesis of neurons. [49]. Furthermore, studies on C. elegans demonstrate a role for MAGI-1 in regulating the clustering of ionotropic glutamate receptors in certain neurons that are associated with learning and memory [13]. Therefore, further characterization of MAGI proteins knockout models is required to determine the role of this subfamily in the CNS.

In summary, the current study completes a series of investigations examining the role of PDZ proteins in regulating the expression, trafficking and signaling of CRFR1. We find that PDZ proteins play an overlapping but distinguishable role in regulating the post-translational modification, ER-Golgi trafficking, endocytosis and signaling of CRFR1 [29,30,31,37]. Although each of these proteins had a similar effect on CRFR1 endocytosis, as they each, with the exception of PDZK1, function to antagonize CRFR1 internalization, they exhibit pleiotropic effects on the regulation of CRFR1-mediated ERK1/2 activity. These observations indicate that there is likely no redundancy of function for PDZ proteins in the regulation of GPCR activity *in vivo*, and suggest that in a cellular context these proteins may interchangeably interact with GPCRs to differentially regulate the recruitment of signaling complexes required for their activation of mitogenic signaling pathways.

## Conclusion

In conclusion, our data demonstrate an interaction between all three MAGI proteins, MAGI-1, MAGI-2 and MAGI-3 with CRFR1 that leads to the regulation of receptor activity. We illustrate that all MAGI proteins can regulate the internalization of the receptor by mediating β-arrestin recruitment. We also provide evidence that MAGI proteins can regulate CRFR1 signaling via the MAPK pathway but not the G protein-dependent pathways presented by cAMP formation. We also suggest a compensatory effect of regulation among the members of the MAGI subfamily. Taken together, these observations along with our previous studies on CRFR1 confirm the distinctive functions of PDZ domain-containing proteins in regulating GPCRs function.

## Abbreviations

ACTH adrenocorticotropin hormone

ANOVA analysis of variance

β_1_AR β_1_-adrenergic receptor

CAL CFTR associated ligand

cAMP cyclic adenosine monophosphate

CNS central nervous system

CRF corticotropin-releasing factor

CRFR1 CRF receptor 1

CRFR2 CRF receptor 2

EPAC exchange proteins directly activated by cAMP biosensor

ERK1/2 extracellular signal-regulated kinase

GPCR G protein-coupled receptor

HA hemagglutinin

HEK293 human embryonic kidney 293

His hexahistidine tagged

HPA axis hypothalamic-pituitary-adrenal axis

LPA_2_R lysophosphatidic acid-activated receptor subtype-2

MAGI Membrane-associated guanylate kinase with inverted orientation PDZ proteins

MAPK mitogen-activated protein kinase

mGluR metabotropic glutamate receptor

PDZ PSD95/Disc Large/Zona Occluden

PSD-95 postsynaptic density protein 95

SAP97 synapse-associated protein 97

VPAC1 Vasoactive Intestinal Polypeptide Type1
